# Regression of central sensitization after bilateral focused ultrasound medial thalamotomies in atypical orofacial pain? – a case report

**DOI:** 10.1186/s12883-026-04966-7

**Published:** 2026-05-18

**Authors:** Matus Velicky Buecheler, Lukasz Kolakowski, Marie Therese Kleinsorge, Heiko Pohl, Lennart Stieglitz, Susanne Wegener

**Affiliations:** 1 Department of Neurology, University Hospital Zurich, Frauenklinikstrasse 26, Zurich, 8091 Switzerland; 2 University of Zurich, Zurich, Switzerland; 3 Department of Neurosurgery, University Hospital Zurich, Zurich, Switzerland

**Keywords:** Orofacial pain, Central sensitization, Focused ultrasound, Central lateral thalamotomy

## Abstract

Orofacial pain syndromes are a heterogenous group of pain disorders resulting in substantial morbidity. They are often refractory to medical treatment and can have an enormous impact on quality of life. Central sensitization plays a major role in chronic pain disorders and poses a diagnostic and therapeutic challenge. Transcranial magnetic resonance-guided focused ultrasound bilateral medial thalamotomy is an emerging treatment in chronic pain syndromes with central sensitization. We here describe a 76-year-old female patient with a long history of atypical orofacial pain and central sensitization with a significant, but temporary regression of the latter after medial thalamotomy using bilateral focused ultrasound.

**Supplementary Information:**

The online version contains supplementary material available at 10.1186/s12883-026-04966-7.

## Introduction

Orofacial pain – *that is pain in the face or mouth* – is one of the most common regional pain disorders [1]. Chronic orofacial pain is a broad term describing unremitting pain syndromes [2] with a large impact on quality of life [3]. However, there are diagnostic and therapeutic uncertainties in this field, delaying proper diagnosis and treatment [4]. The need for a comprehensive classification of orofacial pain was recently met with the publication of the *International Classification of Orofacial Pain*,* 1st Edition (ICOP)* in 2020. Analogous to the ICHD-3, the ICOP offers a clinical tool in navigating the orofacial pain syndromes [4].

Central sensitization (CS) refers to an increase in the excitability of nociceptive neurons [5, 6] and plays a major role both in amplification of pain responses as well as spread of pain to other areas in chronic pain disorders [7]. There is not one *optimal* approach to assess CS [8]. Apart from hyperalgesia and allodynia, expansion of the affected area beyond the initial site of pain is also a characteristic clinical trait of CS [9]. Discriminating between a functional spread of pain due to possible secondary gain and CS can be challenging. Pressure pain thresholds [10], intradermal/topical capsaicin application, low/high-frequency electrical stimulation [8] or somatosensory evoked potentials [11] have been used in assessing CS. Clinical assessment of CS, however, remains a diagnostic and therapeutic challenge with pharmacologic treatments often resulting in unsatisfactory outcomes [12]. Transcranial magnetic resonance-guided focused ultrasound (MRgFUS) has proven its safety and feasibility in refractory neuropathic pain [13–16], rendering it a potential treatment for patients with otherwise intractable chronic pain. This noninvasive stereotactic treatment employs high-intensity focused ultrasound to deliver thermal energy instead of craniotomy, enabling real-time assessment of therapeutic effects and potential adverse events in the awake patient [14].

The following article provides a comprehensive work-up of a case of chronic orofacial pain with CS leading to MRgFUS bilateral thalamotomy, highlighting a novel therapeutic avenue and contributing meaningful evidence to un underexplored field.

## Case report

### History and clinical presentation

A 76-year-old female patient with a 7-year history of COFP in her left maxilla and upper lip was referred for an evaluation of a neurosurgical procedure after having failed several treatments. She had gradually developed the symptoms after extraction of several teeth in her left maxilla for the purpose of fitting a dental prosthesis. She described her pain as burning, pungent and piercing with worsening in a relaxed state and alleviation with physical or psychical distraction. Sleep was profoundly affected. There was no history of neurological or psychiatric disorders.

A thorough diagnostic work-up followed including MRI of the head excluding a neurovascular conflict or other cerebral pathology [Supplemental Fig. 1(a-e)], allergology exam excluding a possible hypersensitivity to metals in the prosthesis and even a tongue biopsy with normal findings. Extensive oral and maxillofacial surgery evaluations failed to identify the exact cause and alleviate the pain. There was no sufficient response to treatment with *pregabalin (maximal dose NA)*,* amitriptyline (up to 35 mg/d)*,* duloxetine (up to 120 mg/d)*,* lamotrigine (up to 150 mg/d)* or *tapentadol (up to 100 mg/d)*. An oral nerve block failed to afford any improvement, neither did an infraorbital-nerve-block or glycerol gangliolysis of the trigeminal ganglion. Her sleep got better with *mirtazapine*, and she reported slight improvement with *gabapentin*, the dose being 3 × 600 mg daily upon referral to our institution.

**Fig. 1:**
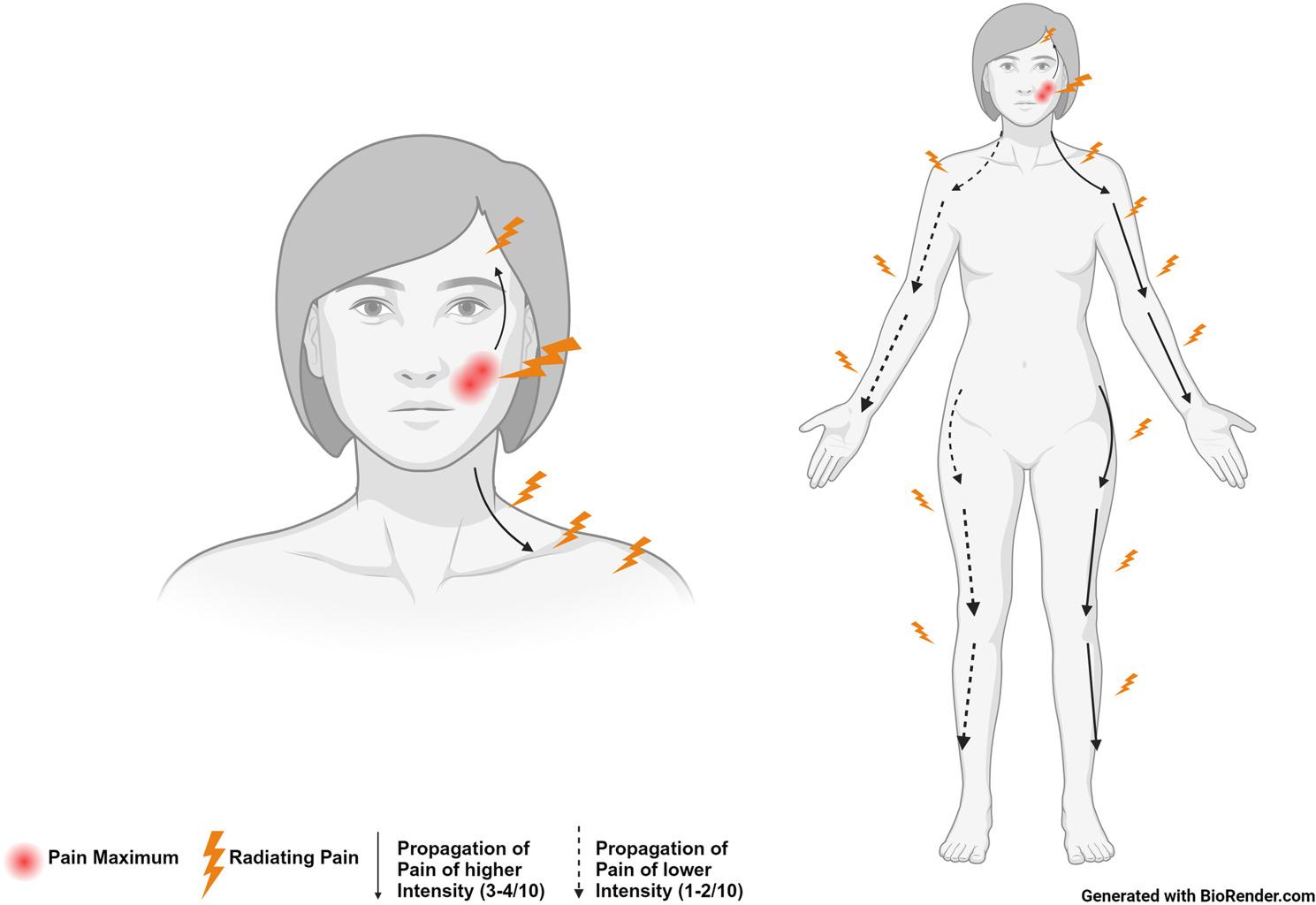
Pain localisation and propagation before bilateral MRgFUS ablation of the CLp. The pain maximum in the maxillary division of the trigeminal nerve on the left is represented in red. The thunderbolts represent radiating pain; the solid lines depict radiating pain of higher intensity; the dashed lines represent radiating pain of lower intensity

Her neurological examination showed a small area of pins-and-needles sensation in the area supplied by the maxillary division of the left trigeminal nerve; both maxillary and mandibular divisions showed slight hypoesthesia *bilaterally* with otherwise normal findings. She located the pain maximum to her left upper lip and maxilla, with propagation to the back of her neck, thorax, and both arms and legs. The pain was constant with the aforementioned qualities and visual analog scale (VAS) of 6/10, with acute exacerbations to 10/10. Pain had spread considerably over the years from being initially localized to the left maxilla and upper lip (Fig. 1).

History and presentation were not consistent with a classical trigeminal neuralgia. Our diagnosis of the pain syndrome remained descriptive, although we could have formally diagnosed *other trigeminal neuropathic pain* according to the ICOP [4]. *Headache attributed to the disorder of the teeth* according to the ICHD-3 remained a differential diagnosis, but this diagnosis was less appropriate considering the extent of the pain syndrome.

### Interdisciplinary assessment

We evaluated the patient in our interdisciplinary board consisting of experts from oral and maxillofacial surgery, neurology, neurosurgery, neuroradiology, ENT, rheumatology, psychiatry and physical therapy. The clinical impression was that central sensitization was now dominating and stereotactic treatment was proposed. Considering the costs and reimbursement from the health insurance, transcranial magnetic resonance-guided focused ultrasound (MRgFUS) was the most suitable option. A pre-operative CT scan showed that the patient technically qualified for the treatment, with a skull density ratio of 0.7. MRI of the brain with navigation protocol followed. The standardized medical and psychiatric evaluation did not reveal contraindications for the stereotactic treatment. Preoperatively the pain assessed using the McGill Pain Questionnaire (MPQ) showed pain rating index (PRI) total of 36/78 (PRI-sensory: 25, -affective: 3, -evaluative: 5, and -miscellaneous: 3), with number of words chosen (NWC) 14, and present pain intensity (PPI) score of 5. The baseline and follow up MPQ values are summarized in Table 1.

**Table 1:** McGill pain questionnaire values as well as visual analog scale at baseline and 2 days and 3- and 6-months post MRgFUS. PRI, Pain rating index; NWC, Number of words chosen; PPI, Present pain intensity, MRgFUS, Transcranial magnetic resonance-guided focused ultrasound

	Baseline	Day 2	3 months	6 Months
PRI-sensory	25	12	13	15
PRI-affective	3	0	0	0
PRI-evaluative	5	0	0	0
PRI-miscellaneous	3	0	6	4
Total PRI	36	12	19	19
NWC	14	5	8	6
PPI	5	1	2	2
VAS	10/10	2/10	3–4/10	4/10

### Neurosurgical procedure

**Fig. 2:**
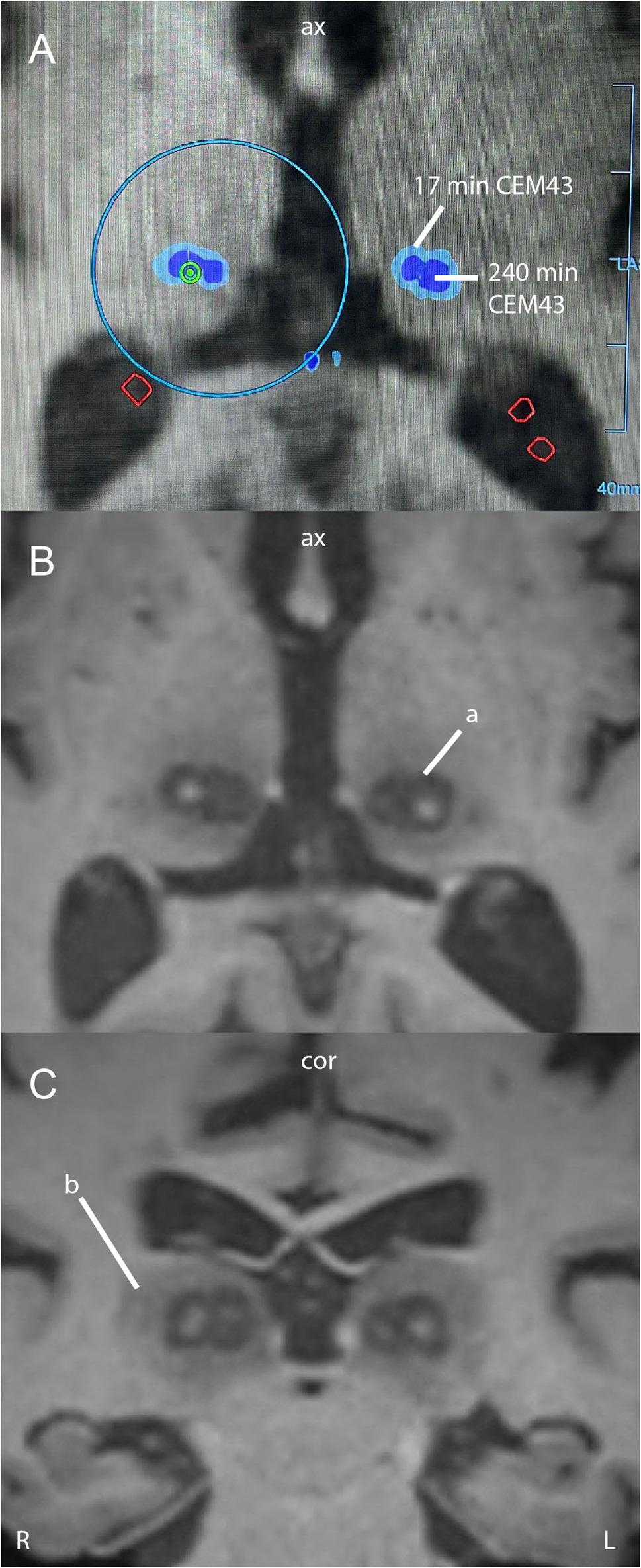
Cranial MRI one day after bilateral MRgFUS ablation of the CLp. **A** Shows a superposition of preoperative MPRAGE with the accumulated CEM43 dose map (cumulative equivalent minutes at 43°C) [17]. The light and dark blue voxels represent the 17 and 240 minutes isodose, respectively. The blue circle illustrates the maximum accessible range of tissue around the transducer focus during the rights-sided ablations. The ablation target during the last sonication is shown in green. The red outlines represent volume with calcified tissue (as typically the choroid plexus), which is excluded from the sonication. **B** and **C** The postoperative MRI (native T1, Siemens 3T Magnetom Vida Fit) on day 1 after the treatment shows the four ablations (a) and the surrounding edema (b)

The patient eventually consented to and underwent MRgFUS of the posterior part of the central lateral nucleus of the thalamus (CLp) bilaterally, 7 years and 2 months after symptom onset (Insightec Exablate 4000 system in combination with Siemens VidaFit 3T MRI). Based on the experience reported by Gallay et al. [15], bilateral ablations were planned from the beginning. She received 4 ablations altogether, 2 per side at 6.5 mm laterally of the ventricular border, 0 mm anterior and 6 mm superior of PC, 9.5 mm laterally, 1.5 mm anterior, 6 mm superior and 5.5 laterally, 0.5 anterior and 6 mm superior, as suggested previously [15] [Fig. 2(a-c)]. Periprocedural medication consisted of 50 mcg of Fentanyl and 4 mg of Ondansetron. The patient was awake during the entire procedure.

Five sonications with ablative intention at 7000 J/sonication were applied altogether, leading to tissue ablation between 56 and 57 °C average, 56–60 °C peak temperature for 11s each. After the first sonication (6.5 mm left) the patient showed a short-term nystagmus to the left, which ceased after seconds without sequelae. After the second sonication (9.5 mm left) the patient reported a mild right-sided facial hypaesthesia, which was completely regredient after 5 min. She reported no immediate effects on the pain until the end of the procedure. The total duration of the procedure was 2:40 h (frame-on to frame-off).

### Post-interventional follow-up

The patient was re-evaluated on the day following the procedure. The local pain intensity in her upper lip/maxilla on the left went from VAS 10/10 just prior to the procedure to VAS 2/10. Strikingly, the pain radiation ceased completely. MPQ on day 2 following the stereotactic treatment yielded a total PRI of 12/78 (sensory 12, affective 0, evaluative 0, and miscellaneous: 0), NWC was 5 and PPI score was 1 (Table 1).

On the follow-up 2 months after the procedure, she could still report a 70% alleviation of the local pain, there was no pain radiation anymore [PRI total of 19/78 (13/0/0/6), NWC 8, PPI 2] (Table 1). The small area of pins-and-needles in the maxillary division of the trigeminal nerve on the left remained, but this was not bothersome for the patient. At 3 months after the treatment her local pain went slightly up (VAS 3–4/10); however, the radiating pain had not come back. Given the good tolerability, the dose of *gabapentin* was increased to 4 × 600 mg daily. The pain had remained well controlled at the 6-month follow up [PRI total of 19/78 (15/0/0/4), NWC 6, PPI 2] (Table 1), there was no impairment of the activities of daily living due to pain reported, and the quality of sleep was reported as very good. The standardized neuropsychological assessment at 6 months demonstrated improvement in the verbal memory as well as the executive functions comparing to baseline. The geriatric depression scale failed to disclose any symptoms of depression.

At the follow-up at 9 months the local pain had remained sufficiently controlled, radiating pain was not reported and gabapentin remained well tolerated. At the follow-up at 17 months the local pain had come back (VAS 6–10/10; MPQ N/A), there was, however, *no radiating* pain in the previously affected areas (Fig. 1). At the last available follow-up at 22 months post MRgFUS bilateral thalamotomy the local pain had persisted (VAS 4–8/10; MPQ N/A), there was, again, some pain radiation in the left arm. There have been no adverse events of the stereotactic treatment reported during the ongoing follow up.

## Discussion

This patient history demonstrates the frequent diagnostic and therapeutic odyssey in people with chronic pain syndromes. Our patient developed a constant and radiating pain beyond the initial symptom presentation over the course of several years with no response from first- to third-line treatments of neuropathic pain [18].

Central sensitization requires a multimodal and individually tailored treatment [19]. The pharmacologic treatment is analogous to treatment of chronic neuropathic pain consisting of tricyclic antidepressants, α2δ ligands and serotonin norepinephrine reuptake inhibitors [19, 20]. However, treatment success remains limited [12].

The role of the central lateral nucleus (CL) of the thalamus, mainly its posterior part (CLp), has long been recognized in maintenance and propagation of *neurogenic pain* and the targeted thalamotomy of CLp has been deemed not only safe, but also effective [15, 16]. The relatively more abundant connections of the CL to the cortex and the spinal cord than the ventral posterior nucleus render this particular target central to the stereotactic suppression of pain [21]. The proposed mechanism in the case presented harnesses the notion that lesion of the dysfunctional CLp in chronic neuropathic pain enables interruption of a self-sustaining thalamocortical pain loop [21–23], rather than influencing the cortical mechanisms of CS directly. In other words, given the nature of the pain syndrome and the lack of relevant comorbidities or factors, which potentially could contribute to maintaining the CS, *bottom-up* [24] subtype of CS was favorized and stereotactic treatment proposed. In a different setting, alternative conservative therapeutic options aiming at cortical spreading (i.E. *top-down* subtype of CS [24]) such as cognitive-behavioral therapy, mindfulness-based stress reduction, transcranial magnetic stimulation or ketamine infusions, to name just a few, might have been tried.

Emerging evidence suggests that MRgFUS is a safe, feasible and effective treatment in chronic neuropathic pain [13–16, 25]. The reported pain syndromes treated with MRgFUS so far consisted mostly of unilateral or localized neuropathic pain or neuralgia of a known cause. The focus of this case report is on the effects of MRgFUS *beyond the areas* of initial neuropathic pain. In our patient, not only did the MRgFUS significantly alleviate the local neuropathic pain, it abolished the radiating pain to otherwise healthy body parts, thus further supporting the clinical impression that CS had played a major role in this particular pain syndrome. Given the nature of this report, the lack of auxiliary testing regarding CS, and pain resurgence after 17 months, we cannot differentiate between a reduction of central sensitization or a temporary, complete reversal. Should future research corroborate our observation, MRgFUS could become an experimental treatment option in chronic pain syndromes with CS. Pain had remained adequately controlled for more than 1 year after the stereotactic treatment but recurred before the 17-month follow-up.

The major limitation of this case report is the limited follow-up. Second, we have not utilized auxiliary assessments in regard to CS pre-/post intervention and thus provide solely the clinical impression. Third, alternative conservative therapeutic approaches to CS had not been applied, as these were deemed less promising given the setting. Fourth, we cannot completely rule out medication confounding as well as placebo effect but deem both less likely, given the time course of pain alleviation. Given the temporary effect and pain resurgence, more long-term data is warranted to be able to draw conclusions in regard to MRgFUS in the treatment of chronic pain with CS.

## Conclusion

Transcranial magnetic resonance-guided focused ultrasound poses a promising investigational treatment modality in otherwise intractable neuropathic pain syndromes with central sensitization, however, long-term data are warranted given the possible temporary effect.

## Supplementary Information

Supplementary Material 1: Preoperative cranial MRI. T2 weighted 3D Space sequence in transversal plane (1a, slice thickness 0.3mm) and coronal plane (1b, slice thickness 0.35mm) at the level of the root entry zone (REZ) of the trigeminal nerve. 1c: Red arrow on the right showing close spatial relationship of the REZ to the superior cerebellar artery (SCA) without clear vascular conflict. Blue arrow on the left shows no notable conflict. There was no relevant vascular conflict reported be the neuroradiologist in the serial preoperative MRIs. T2 weighted fat-suppressed sequence (1d) and T1 weighted post-gadolinium sequence (1e) in transversal plane showing normal brain parenchyma at the REZ bilaterally.

## Data Availability

No new data was generated in this work. Patient privacy considerations preclude public sharing of the data supporting this case report. Further enquiries can be directed to the corresponding author.

## Abbreviations

CLp: Central lateral nucleus of the thalamus, posterior part
CS: Central sensitization
ICHD-3: International classification of headache disorders, 3^rd^ edition
MRgFUS: Transcranial magnetic resonance-guided focused ultrasound
