# Supplementary figures and images for: Regression of central sensitization after bilateral focused ultrasound medial thalamotomies in atypical orofacial pain? – a case report
Source: BMC Neurol. 2026 May 18;26:444. doi: 10.1186/s12883-026-04966-7 (PMC13348938; doi:10.1186/s12883-026-04966-7)

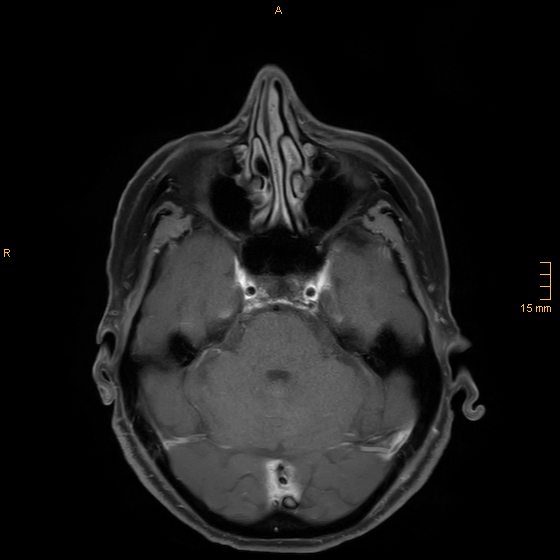

Supplement: Supplementary file 1 — Supplementary Material 1: Preoperative cranial MRI. T2 weighted 3D Space sequence in transversal plane (1a, slice thickness 0.3mm) and coronal plane (1b, slice thickness 0.35mm) at the level of the root entry zone (REZ) of the trigeminal nerve. 1c: Red arrow on the right showing close spatial relationship of the REZ to the superior cerebellar artery (SCA) without clear vascular conflict. Blue arrow on the left shows no notable conflict. There was no relevant vascular conflict reported be the neuroradiologist in the serial preoperative MRIs. T2 weighted fat-suppressed sequence (1d) and T1 weighted post-gadolinium sequence (1e) in transversal plane showing normal brain parenchyma at the REZ bilaterally. [file 12883_2026_4966_MOESM1_ESM.zip › Complete Regression of Central Sensitization after Bilateral Focused Ultrasound Medial Thalamotomies in Atypical Orofacial Pain - Supplemental Figure 1e.tiff]

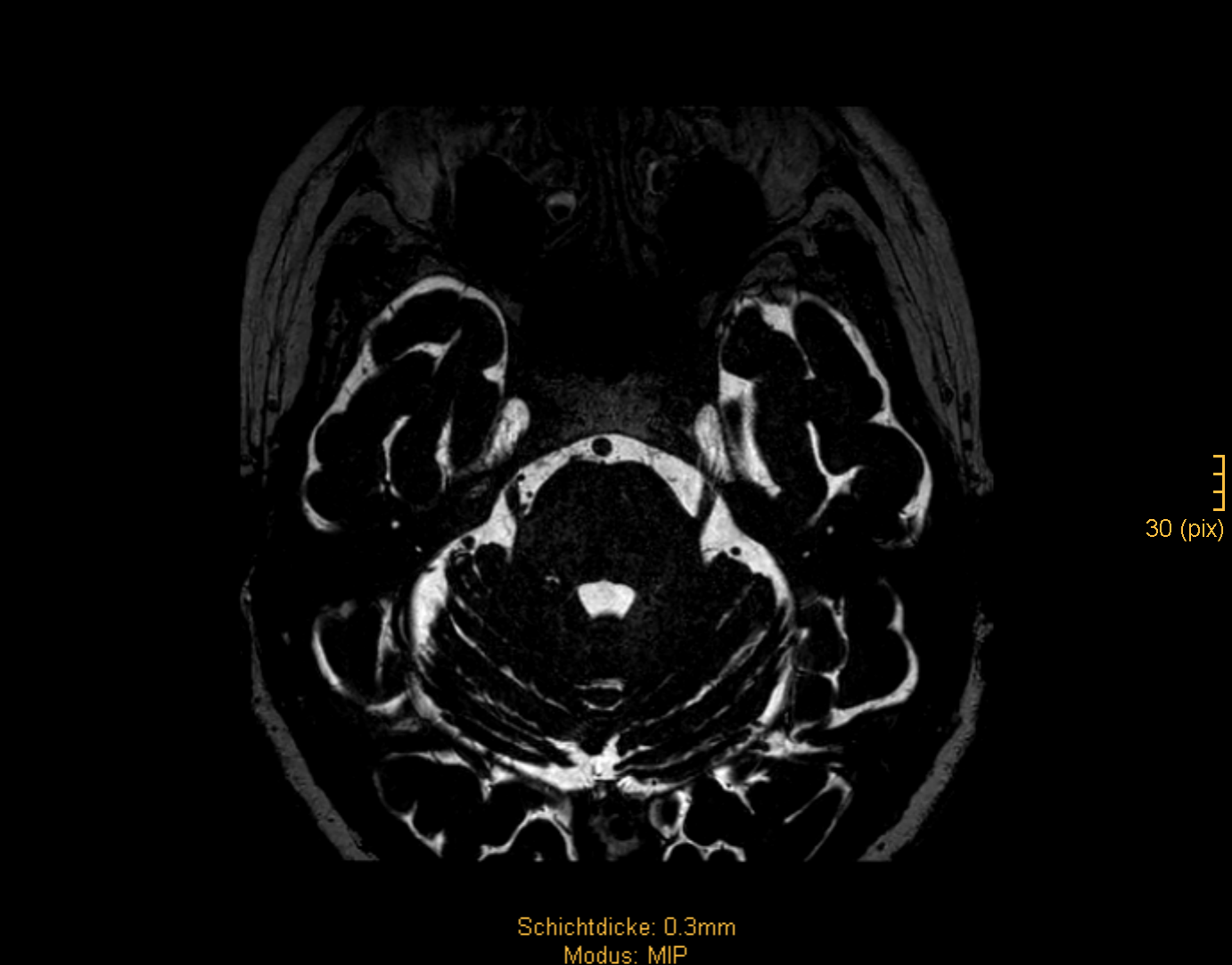

Supplement: Supplementary file 1 — Supplementary Material 1: Preoperative cranial MRI. T2 weighted 3D Space sequence in transversal plane (1a, slice thickness 0.3mm) and coronal plane (1b, slice thickness 0.35mm) at the level of the root entry zone (REZ) of the trigeminal nerve. 1c: Red arrow on the right showing close spatial relationship of the REZ to the superior cerebellar artery (SCA) without clear vascular conflict. Blue arrow on the left shows no notable conflict. There was no relevant vascular conflict reported be the neuroradiologist in the serial preoperative MRIs. T2 weighted fat-suppressed sequence (1d) and T1 weighted post-gadolinium sequence (1e) in transversal plane showing normal brain parenchyma at the REZ bilaterally. [file 12883_2026_4966_MOESM1_ESM.zip › Complete Regression of Central Sensitization after Bilateral Focused Ultrasound Medial Thalamotomies in Atypical Orofacial Pain - Supplemental Figure 1a.tiff]

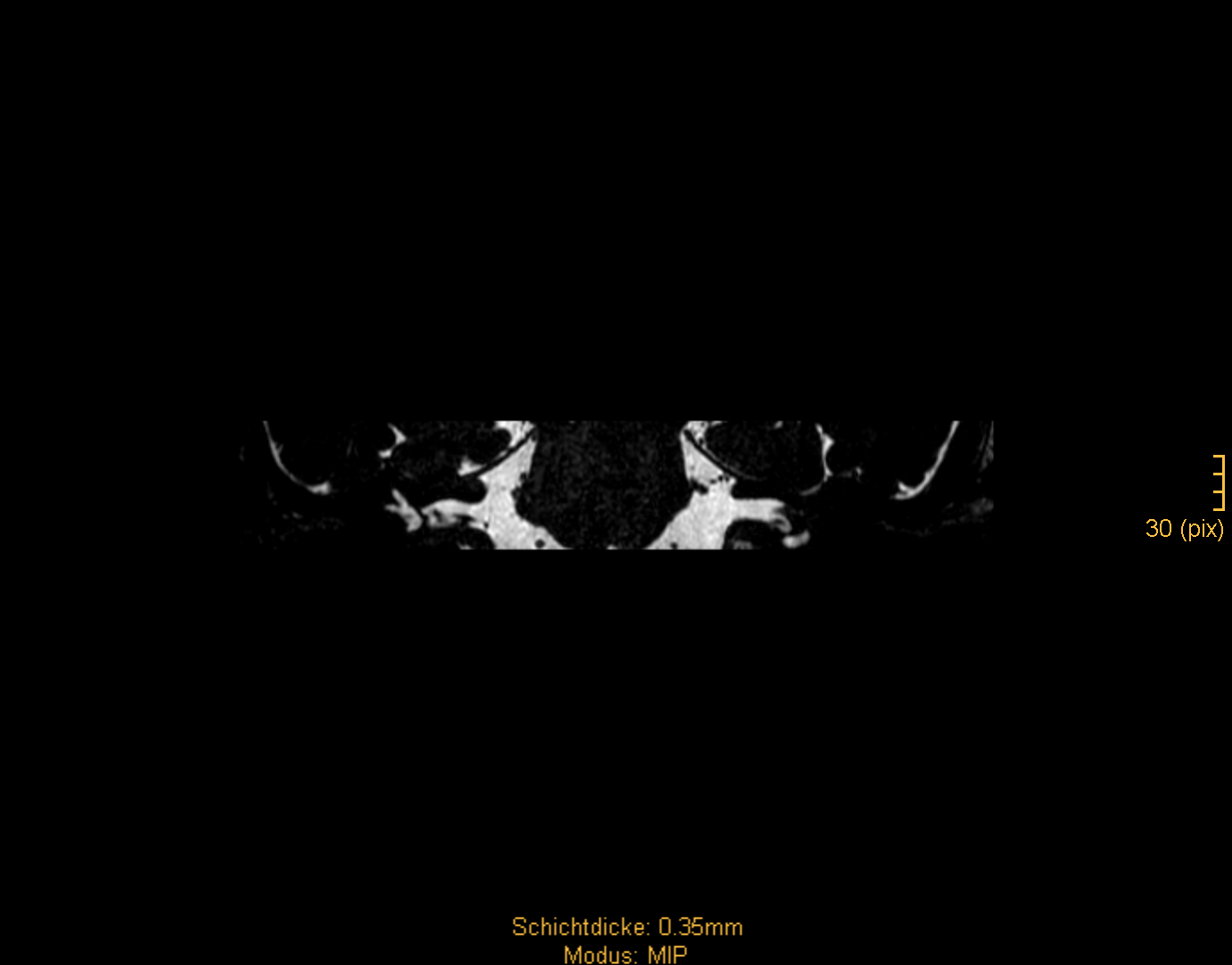

Supplement: Supplementary file 1 — Supplementary Material 1: Preoperative cranial MRI. T2 weighted 3D Space sequence in transversal plane (1a, slice thickness 0.3mm) and coronal plane (1b, slice thickness 0.35mm) at the level of the root entry zone (REZ) of the trigeminal nerve. 1c: Red arrow on the right showing close spatial relationship of the REZ to the superior cerebellar artery (SCA) without clear vascular conflict. Blue arrow on the left shows no notable conflict. There was no relevant vascular conflict reported be the neuroradiologist in the serial preoperative MRIs. T2 weighted fat-suppressed sequence (1d) and T1 weighted post-gadolinium sequence (1e) in transversal plane showing normal brain parenchyma at the REZ bilaterally. [file 12883_2026_4966_MOESM1_ESM.zip › Complete Regression of Central Sensitization after Bilateral Focused Ultrasound Medial Thalamotomies in Atypical Orofacial Pain - Supplemental Figure 1b.tiff]

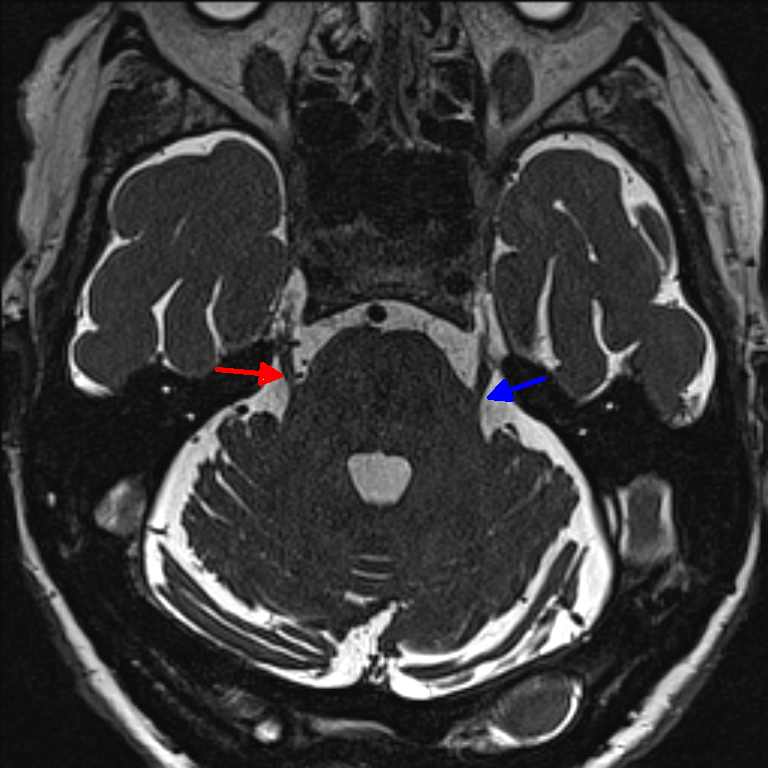

Supplement: Supplementary file 1 — Supplementary Material 1: Preoperative cranial MRI. T2 weighted 3D Space sequence in transversal plane (1a, slice thickness 0.3mm) and coronal plane (1b, slice thickness 0.35mm) at the level of the root entry zone (REZ) of the trigeminal nerve. 1c: Red arrow on the right showing close spatial relationship of the REZ to the superior cerebellar artery (SCA) without clear vascular conflict. Blue arrow on the left shows no notable conflict. There was no relevant vascular conflict reported be the neuroradiologist in the serial preoperative MRIs. T2 weighted fat-suppressed sequence (1d) and T1 weighted post-gadolinium sequence (1e) in transversal plane showing normal brain parenchyma at the REZ bilaterally. [file 12883_2026_4966_MOESM1_ESM.zip › Complete Regression of Central Sensitization after Bilateral Focused Ultrasound Medial Thalamotomies in Atypical Orofacial Pain - Supplemental Figure 1c.tif]

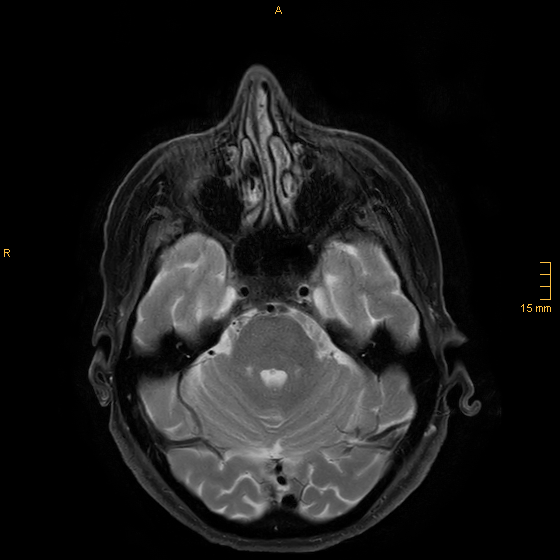

Supplement: Supplementary file 1 — Supplementary Material 1: Preoperative cranial MRI. T2 weighted 3D Space sequence in transversal plane (1a, slice thickness 0.3mm) and coronal plane (1b, slice thickness 0.35mm) at the level of the root entry zone (REZ) of the trigeminal nerve. 1c: Red arrow on the right showing close spatial relationship of the REZ to the superior cerebellar artery (SCA) without clear vascular conflict. Blue arrow on the left shows no notable conflict. There was no relevant vascular conflict reported be the neuroradiologist in the serial preoperative MRIs. T2 weighted fat-suppressed sequence (1d) and T1 weighted post-gadolinium sequence (1e) in transversal plane showing normal brain parenchyma at the REZ bilaterally. [file 12883_2026_4966_MOESM1_ESM.zip › Complete Regression of Central Sensitization after Bilateral Focused Ultrasound Medial Thalamotomies in Atypical Orofacial Pain - Supplemental Figure 1d.tiff]
